# Sleep Disorders in Parkinsonian and Nonparkinsonian *LRRK2* Mutation Carriers

**DOI:** 10.1371/journal.pone.0132368

**Published:** 2015-07-15

**Authors:** Claustre Pont-Sunyer, Alex Iranzo, Carles Gaig, Ana Fernández-Arcos, Dolores Vilas, Francesc Valldeoriola, Yaroslau Compta, Ruben Fernández-Santiago, Manel Fernández, Angels Bayés, Matilde Calopa, Pilar Casquero, Oriol de Fàbregues, Serge Jaumà, Victor Puente, Manel Salamero, Maria José Martí, Joan Santamaría, Eduard Tolosa

**Affiliations:** 1 Parkinson’s Disease and Movement Disorders Unit, Neurology Service, Hospital Clinic de Barcelona, Universitat de Barcelona, Institut d’Investigacions BiomediquesAugust Pi I Sunyer (IDIBAPS), Centro de Investigación Biomédica en Red sobre Enfermedades Neurodegenerativas (CIBERNED), Barcelona, Spain; 2 MultidisciplinarySleepDisordersUnit, Neurology Service, Hospital Clinic de Barcelona, Universitat de Barcelona, Institut d’Investigacions BiomediquesAugust Pi I Sunyer (IDIBAPS), Centro de Investigación Biomédica en Red sobre Enfermedades Neurodegenerativas (CIBERNED), Barcelona, Spain; 3 Laboratory of Neurodegenerative Disorders, Department of Clinical and Experimental Neurology, Institut d’Investigacions Biomèdiques August Pi i Sunyer (IDIBAPS), Hospital Clínic de Barcelona, Universitat de Barcelona, Centro de Investigación Biomédica en Red sobre Enfermedades Neurodegenerativas (CIBERNED), Barcelona, Spain; 4 Unidad de Parkinson Teknon, Barcelona, Spain; 5 NeurologyService, Hospital Universitari de Bellvitge, Barcelona, Spain; 6 Hospital Mateu Orfila, Maó, Menorca, Spain; 7 Neurology Service, Hospital Universitari Vall D’Hebron, Barcelona, Spain; 8 Neurology Service, Hospital Del Mar, Barcelona, Spain; 9 PsychologyService, Hospital Clinic,Barcelona, Spain; University of Rome Tor Vergata, ITALY

## Abstract

**Objective:**

In idiopathic Parkinson disease (IPD) sleep disorders are common and may antedate the onset of parkinsonism. Based on the clinical similarities between IPD and Parkinson disease associated with *LRRK2* gene mutations (LRRK2-PD), we aimed to characterize sleep in parkinsonian and nonmanifesting *LRRK2* mutation carriers (NMC).

**Methods:**

A comprehensive interview conducted by sleep specialists, validated sleep scales and questionnaires, and video-polysomnography followed by multiple sleep latency test (MSLT) assessed sleep in 18 LRRK2-PD (17 carrying G2019S and one R1441G mutations), 17 NMC (11 G2019S, three R1441G, three R1441C), 14 non-manifesting non-carriers (NMNC) and 19 unrelated IPD.

**Results:**

Sleep complaints were frequent in LRRK2-PD patients; 78% reported poor sleep quality, 33% sleep onset insomnia, 56% sleep fragmentation and 39% early awakening. Sleep onset insomnia correlated with depressive symptoms and poor sleep quality. In LRRK2-PD, excessive daytime sleepiness (EDS) was a complaint in 33% patients and short sleep latencies on the MSLT, which are indicative of objective EDS, were found in 71%. Sleep attacks occurred in three LRRK2-PD patients and a narcoleptic phenotype was not observed. REM sleep behavior disorder (RBD) was diagnosed in three LRRK2-PD. EDS and RBD were always reported to start after the onset of parkinsonism in LRRK2-PD. In NMC, EDS was rarely reported and RBD was absent. When compared to IPD, sleep onset insomnia was more significantly frequent, EDS was similar, and RBD was less significantly frequent and less severe in LRRK2-PD. In NMC, RBD was not detected and sleep complaints were much less frequent than in LRRK2-PD. No differences were observed in sleep between NMC and NMNC.

**Conclusions:**

Sleep complaints are frequent in LRRK2-PDand show a pattern that when compared to IPD is characterized by more frequent sleep onset insomnia, similar EDS and less prominent RBD. Unlike in IPD, RBD and EDS seem to be not markers of the prodromal stage of LRRK2-PD.

## Introduction

Idiopathic Parkinson disease (IPD) is a neurodegenerative condition where neuronal loss in the substantia nigra leads to motor symptoms. Patients with IPD also present a variety of nonmotor symptoms such as sleep disorders, hyposmia, depression, constipation, hallucinations and dementia. Damage of brain structures outside the substantia nigra is the origin of certain nonmotor symptoms and in some cases they may antedate parkinsonism onset in what has been called the prodromal phase of PD [1].

Sleep disorders such as insomnia, excessive daytime sleepiness (EDS) and REM sleep behavior disorder (RBD) are common in IPD. RBD, and possibly EDS, may antedate the onset of parkinsonism in IPD. The origin of sleep disturbances in IPD is multifactorial and includes damage of the brain structures that modulate sleep, the coexistence of symptoms that may disrupt sleep onset and maintenance (e.g., depression, nocturnal immobility), and the effect of some medications (e.g., dopaminergic agents, antidepressants). Identification and management of sleep disorders in IPD is important since they frequently have a negative impact on quality of life [2–4].

Mutations in the leucine-rich repeat kinase 2 gene (LRRK2) are associated with sporadic and familial PD (LRRK2-PD)[5–6]. Penetrance of LRRK2 mutations is incomplete and age-dependent, and therefore a proportion of asymptomatic individuals carrying mutations can be at the premotor stage of LRRK2-PD[7]. When manifested, clinical expression of LRRK2-PD is usually similar to IPD and consists of late onset levodopa-responsive parkinsonism, typical complications of dopaminergic therapy, and nonmotor features including hyposmia and depression[8–15].

Available data on sleep disturbances in individuals carrying *LRRK2* mutations is scarce. Based on the clinical similarities between IPD and LRRK2-PD we hypothesized that those sleep disorders occurring in IPD may also be present in LRRK2-PD. Also, we hypothesized that sleep disorders that are known to occur in premotor IPD may also be present in LRRK2 mutation carriers who have not developed parkinsonism yet. In the current study we sought to characterize the sleep disturbances occurring in parkinsonian and nonparkinsonian*LRRK2* mutation carriers through subjective and objective measures of sleep. For comparative purposes, sleep data on *LRRK2* mutation carriers were compared with relatives who were non carriers of LRRK2 mutations and with unrelated IPD patients.

## Methods

### Participants and genetic assessment

The current study comprises individuals in whom the assessment of the *LRRK2* genetic status was determined at the Hospital Clinic de Barcelona, Barcelona, Spain. The study was conducted between January 2012 and September 2013 in the Hospital Clinic de Barcelona.

Sixty-eight subjects were consecutively enrolled in the study, irrespective for sleep complaints. We included the following groups for evaluations and comparisons: 1) 18PD carriers of *LRRK2* mutations (LRRK2-PD), 2) 17 first-degree relatives of LRRK2-PD patients who were carriers of LRRK2 mutations and had no parkinsonism (non-manifesting carriers, NMC) and, 3) 14 relatives of LRRK2-PD patients who were negative for LRRK2 mutations and had no parkinsonism (non-manifesting non-carriers, NMNC). During routine visits at the movement disorders clinic, we recruited a group of 19 IPD patients who were consecutively selected to match the LRRK2-PD group in age, gender, disease duration and Hoehn and Yahr stage[16].

Diagnosis of PD was made according the UK Brain Bank Criteria[17]. Exclusion criteria in LRRK2-PD and IPD groups were age at parkinsonism onset less than 30 years, Hoehn and Yahr stage ≥4[16], dementia[18], and therapy with deep brain stimulation, apomorphine continuous infusionor intestinal levodopa gel infusion. Exclusion criteria in the NMC and NMNC groups were age less than 18 years, and presence of motor symptoms, a neurological disorder or a disabling disease.

Genotyping of G2019S, R1441G and R1441C mutations in the *LRRK2* gene was performed on blood samples as previously described[9]. Patients with IPD were negative for mutations in *LRRK2* gene. LRRK2-PD patients were aware of their genetic status. Subjects in the NMC and NMNC groups were unaware of their genetic status.

In each participant all clinical and sleep assessments were done during a 2-day period. The ethical committee at our institution approved the study and all participants gave written informed consent.

### Clinical assessment

Movement disorders expert neurologists (CPS and DV) conducted a medical interview that included demographic and clinical data, and current medications including calculation of total L-Dopa equivalent daily dose[19]. At the time of assessment all drugs had been stable during the previous month. Motor and non-motor features, as well as their impact on daily living activities, were evaluated with the Movement Disorders Society Unified Parkinson Disease Rating Scale (MDS-UPDRS parts I to IV)[20], Hoehn and Yahr scale[16], and Schwab and England activities of daily living scale[21]. Motor phenotype subtype was classified in postural instability and gait difficulty, tremor dominant, and indeterminate[22]. The impact of PD upon quality of life was determined with the PD questionnaire (PDQ-39)[23]and anxiety and depression by means of the Hospital Anxiety and Depression scale (HADS)[24]. Cognition was evaluated using the Montreal Cognitive Assessment (MoCA)[25]and a score of less than 21 points was suggestive of cognitive impairment (according to a cut-off score established in Spanish population)[26].

### Sleep assessment

Sleep-related symptoms, video-polysomnography (V-PSG) and multiple sleep latency test (MSLT) were evaluated by sleep specialists (AFA, CG, AI and JS) who were blind to the participant category and genetic status (LRRK2-PD, IPD, NMC or NMNC). Sleep evaluation included the following assessments:

#### 1. Sleep-related symptomatology

This was examined with a comprehensive semi-structured sleep interview conducted by sleep experts where the presence of the bed partner or anyone who witnessed the patient’s sleep was encouraged to substantiate and complete the patient sleep history. The interview covered sleep habits and complaints such as sleep onset insomnia (difficulty falling asleep within 30 minutes or more), sleep fragmentation (more than two awakenings lasting more than 15 minutes), early awakening (waking up at least two hours earlier than desired), nocturnal akinesia (difficulty in turning over in bed), excessive daytime sleepiness (EDS, inability to stay awake during the major periods of the day resulting in unintended episodes of sleep), sleep attacks (episodes of sudden onset of sleep without warning),nightmares and dream-enacting behaviors. According to the sleep interview these sleep complaints were categorized as being present or absent in each participant. In addition, overall sleep disturbances and nocturnal disability were evaluated with the Pittsburgh Sleep Quality Index (PSQI)[27] and the modified Parkinson’s Disease Sleep Scale (PDSS-2)[28]. PSQI global score ≥6 points and PDSS-2 total score ≥15 were indicative of poor sleep quality[27, 29].

Subjective EDS was also assessed by means of the Epworth Sleepiness Scale (ESS), where a score ≥11 points was suggestive of EDS[30]. Sleep attacks were also identified using the Inappropriate Sleep Composite Score (ISCS).[31].

Restless legs syndrome (RLS) was diagnosed according to the International Restless Legs Syndrome Study Group criteria[32]. Individuals who fulfilled the four diagnostic criteria for RLS were then interviewed to verify its presence and exclude potential RLS mimics (e.g., stiffness, cramps, off periods). RLS severity was measured with the International RLS Study Group Rating Scale[33].

REM sleep behavior disorder (RBD) was diagnosed according to the International Classification of sleep disorders 3^rd^ revision criteria (ICSD-III)[34]. In brief, RBD was diagnosed in patients with a chronic history of dream-enacting behaviors (reported by the patient or the bed partner) in whom video-polysomnography (V-PSG) demonstrated excessive electromyographic (EMG) activity in REM sleep (see below for V-PSG definition of “excessive EMG activity”) linked to abnormal vigorous movements. RBD was also diagnosed in patients who were unaware of displaying dream-enacting behaviors but V-PSG demonstrated excessive EMG activity in REM sleep associated with vigorous behaviors that are typical of RBD (e.g., prominent body jerking, kicking, screaming)[34]. Besides, the RBD screening questionnaire (RBDSQ) was administrated to assess its usefulness to detect true RBD in *LRRK2* mutation carriers[35]. A RBDSQ score ≥5 in nonparkinsonian subjects and a RBDSQ score ≥7 in parkinsonian subjects has been considered suggestive of RBD[36].

#### 2. Video-polysomnography (V-PSG)

This was performed in all participants with a digital polygraph (Deltamed, software version 2007, Paris, France) and consisted of electroencephalogram (F3, F4, C3, C4, O1, and O2, referred to the contralateral ear), right and left electro-oculograms, surface EMG of the mentalis muscle, surface EMG of the right and left flexor digitorum superficialis in the upper limbs, surface EMG of the right and left anterior tibialis in the lower limbs, electrocardiogram, nasal and oral air-flow assessment, nasal pressure cannula, thoracic and abdominal movement assessment, and measurement of oxyhemoglobin saturation. Sleep stages were scored according to standard criteria with the allowance for REM sleep without atonia[37]. Apneas, hypopneas, arousals and periodic leg movements in sleep (PLMS) were scored according to standard criteria[37]. The apnea/hypopnea index (AHI) was defined as the mean number of apneas and hypopneas per hour of sleep, the arousal index was defined as the mean number of arousals per hour of sleep, and the PLMS index (PLMSI) was defined as the number of PLMS per hour of sleep[37].

For the measurement of EMG activity in REM sleep we followed the SINBAR method[38] where a trained neurologist (AFA) visually quantified “any” (tonic, phasic or a combination of both) EMG activity in the mentalis muscle plus phasic EMG activity in the right and left flexor digitorum superficialis muscles. Following this method, ≥32% of 3-sec mini-epochs containing EMG activity in REM sleep was considered “excessive EMG activity” and indicative of RBD[38].

#### 3. Multiple SleepLatency Test (MSLT)

This test evaluated the presence of objective EDS. The MSLT was performed according to standard criteria and consisted of five naps at 2-hour intervals beginning two hours after the end of the V-PSG[39]. A mean sleep latency onset of ≤8 min was considered indicative of objective EDS[34]. A mean sleep latency of ≤8 min plus more than one sleep onset period (SOREMP) defined a narcoleptic phenotype[3].

### Statistical analysis

Data are reported in mean, standard deviation, number and percentage. Quantitative variables were analyzed using Student’s t-test for comparisons of two independent groups. Qualitative variables were analyzed using χ2-test and the Fisher exact test, when appropriate. No imputation was made for missing data. Subjects missing values in a particular field were not included in the analysis for that particular outcome. Secondary analysis controlling for confounders like sleep-related drug intake (e.g., benzodiazepines, dopamine agents) was performed by means of ANOVA or logistic regression in case of differences between groups. Pearson’s correlation coefficients were used to examine in the LRRK2-PD group the association between variables and sleep onset insomnia and a mean sleep latency onset ≤8 on the MSLT. Two tailed P values of less than 0.05 were considered to be significant. Statistical analyses were performed with SPSS version 20.0 (IBM).

## Results

The study comprised 18 LRRK2-PD subjects from 17 families (17 patients carrying the G2019S mutation and one the R1414G mutation), 17 NMC from 13 families (11 G2019S, three R1414G and three R1414C), 14 NMNC from nine families (11 relatives of LRRK2-PD patients carrying the G1920S mutation, and three individuals from the same family of a R1441G LRRK2-PD patient), and 19 unrelated IPD patients. All seven subjects with the R1441G mutation (one LRRK2-PD, three NMC and three NMNC) were from the same family. The three NMC subjects carrying the R1441C mutation were siblings. All participants included in this study were Caucasian Spanish except one G2019S LRRK2-PD subject of North-African origin. All participants lived in Northeast Spain (63 in Catalonia and five in Menorca). Demographic, parkinsonian and sleep related clinical data, V-PSG data and comparisons between groups are presented in Tables 1, 2 and 3.

**Table 1:** Demographic and parkinsonian clinical data and comparisons between groups.

	LRRK2-PD(n = 18)	IPD(n = 19)	NMC(n = 17)	NMNC(n = 14)	*p* value(LRRK2-PD vs. IPD)	*p* value(LRRK2-PD vs. NMC)	*p* value(NMC vs. NMNC)
**Age (years)**	61.0 ± 11.2	63.1 ± 11.2	44.4 ± 12.3	50.8 ± 16.0	0.996	<0.001	0.557
**Female gender n (%)**	9 (50.0)	8 (42.1)	9 (52.3)	7 (50.0)	0.758	0.849	0.858
**Body mass Index (Kg/m^2^)**	26.4 ± 3.7	26.4 ± 2.8	25.8 ± 2.7	24.4 ± 3.8	0.966	0.677	0.299
**Age at PD diagnosis (years)**	53.2 ± 10.2	55.9 ± 11.2	NA	NA	0.440	NA	NA
**PD duration (years)**	7.8 ± 5.8	7.3 ± 3.7	NA	NA	0.746	NA	NA
**L-dopa intake n (%)**	11 (61.1)	11 (57.9)	0 (0.0)	0 (0.0)	0.860	NA	NA
**Dopaminergic agonist intake n (%)**	16 (88.8)	11 (57.9)	0 (0.0)	0 (0.0)	0.021	NA	NA
**L-Dopa daily equivalent dose (mg)**	871.4 ± 669.2	550.2 ± 478.9	0 ± 0	0 ± 0	0.109	NA	NA
**Antidepressants intake n (%)**	4 (22.2)	4 (21.0)	2 (11.7)	2 (14.3)	0.867	0.510	0.941
**Benzodiazepine intake n (%)**	10 (55.5)	2 (10.5)	1 (5.8)	3 (21.4)	0.003	0.003	0.249
**UPDRS-I score (n)**	10.2 ± 7.4	12.3 ± 7.6	3.1 ± 4.2	3.0 ± 4.0	0.398	0.002	0.931
**UPDRS- II score (n)**	8.9 ± 5.2	11.6 ± 7.9	0.2 ± 0.4	0.3 ± 0.5	0.239	<0.001	0.605
**UPDRS-III score (n)**	14.4 ± 7.7	21.8 ± 10.8	1.1 ± 1.9	0.2 ± 0.4	0.022	<0.001	0.085
**UPDRS-IV score (n)**	2.3 ± 3.5	1.4 ± 2.3	0 ± 0	0 ± 0	0.329	0.015	NA
PIGD subtype n (%)	10 (55.5)	10 (52.6)	NA	NA	0.756	NA	NA
Indeterminate subtype n (%)	4 (22.2)	1 (5.3)	NA	NA			
Tremor dominant subtype n (%)	4 (22.2)	4 (21.0)	NA	NA			
**Hoehn and Yahr stage (n)**	2.0 ± 0.7	1.9 ± 0.6	0 ± 0	0 ± 0	0.931	0.036	0.600
**Schwab and England score (n)**	86.1 ± 10.4	87.0 ± 10.8	100 ± 0	100 ± 0	0.797	<0.001	NA
**PDQ-39 score (n)**	35.9 ± 15.2	36.2 ± 23.3	5.5 ± 6.4	7.4 ± 9.4	0.960	<0.001	0.544
**HADS total score (n)**	12.2 ± 6.1	10.5 ± 4.7	8.9 ± 5.8	8.3 ± 6.7	0.346	0.116	0.804
**HADS-anxiety score (n)**	6.8 ± 3.8	6.4 ± 2.3	5.9 ± 3.5	5.6 ± 4.3	0.658	0.491	0.806
**HADS-depression score (n)**	5.4 ± 3.2	4.3 ± 2.9	2.9 ± 2.5	2.8 ± 3.1	0.277	0.023	0.890
**MoCA score (n)**	24.2 ± 4.7	24.9 ± 2.4	26.3 ± 2.4	27.4 ± 1.9	0.714	0.108	0.192

Data are presented as mean, standard deviation, number and percentage.

**HADS**: Hospital Anxiety and Depression scale; **IPD**: Idiopathic Parkinson Disease; **LRRK2-PD**: LRRK2 Parkinson Disease; **MoCA**: Montreal Cognitive Assessment; **NA**: Not applicable; **NMC**: Non-manifesting carriers; **NMNC:** Non-manifesting non-carriers; **PDQ-39**: 39-item Parkinson Disease Questionnaire; **PIGD**: postural instability gait difficulty; **PD**: Parkinson disease; **UPDRS**: Unified Parkinson Disease Rating Scale.

**Table 2:** Sleep related clinical data and comparisons between groups.

	LRRK2-PD(n = 18)	IPD(n = 19)	NMC(n = 17)	NMNC(n = 14)	*p* value(LRRK2-PD vs. IPD)	*p* value(LRRK2-PD vs. NMC)	*p* value(NMC vs. NMNC)
**PSQI score (n)**	8.3 ± 3.5	5.7 ± 3.6	3.6 ± 3.3	4.4 ± 4.8	0.033	<0.001	0.590
**PSQI score ≥6 n (%)**	14 (77.7)	8 (42.1)	1 (5.8)	3 (21.4)	0.025	<0.001	0.330
**PDSS-2 score (n)**	14.4 ± 7.7	10.2 ± 6.9	6.3 ± 6.2	3.4 ± 6.2	0.102	0.003	0.226
**PDSS-2 score ≥15 n (%)**	8 (44.4)	6 (31.6)	2 (11.7)	1 (7.1)	0.486	0.060	1.00
**Sleep onset insomnia n (%)**	6 (33.3)	0 (0.0)	2 (11.7)	3 (21.4)	0.005	0.242	0.564
**Sleep fragmentation n (%)**	10 (55.5)	5 (26.3)	4 (23.5)	2 (14.3)	0.054	0.158	0.411
**Early awakening n (%)**	7 (38.8)	7 (36.8)	1 (5.8)	2 (14.3)	0.804	0.046	0.501
**Nocturia n (%)**	11 (61.1)	12 (63.1)	10 (58.8)	6 (42.9)	0.944	1.000	0.198
**Nocturnal tremor n (%)**	2 (11.1)	3 (15.8)	0 (0.0)	0 (0.0)	0.723	0.489	NA
**Nocturnal rigidity n (%)**	5 (27.7)	3 (15.8)	0 (0.0)	0 (0.0)	0.335	0.049	NA
**Nocturnal akinesia n (%)**	9 (50.0)	9 (47.4)	0 (0.0)	0 (0.0)	0.758	0.001	NA
**Early morning dystonia n (%)**	4 (22.2)	1 (5.3)	0 (0.0)	0 (0.0)	0.117	0.108	NA
**Restless legs syndrome (%)**	3 (16.6)	3 (15.8)	2 (11.7)	2 (14.3)	0.911	1.000	0.941
**IRLSGRS score (n)**	4.0 ± 8.0	1.0 ± 4.0	2.0 ± 5.0	2.0 ± 6.0	0.505	0.723	0.382
**Excessive daytime sleepiness complaint n (%)**	6 (33.3)	9 (47.3)	3 (17.6)	0 (0)	0.463	0.442	0.077
**ESS score (n)**	7.8 ± 5.0	8.3 ± 2.7	7.3 ± 4.5	5.5 ± 2.2	0.756	0.766	0.171
**ESS score ≥11 n (%)**	4 (22.2)	5 (26.3)	4 (23.5)	0 (0.0)	1.00	1.000	0.100
**Sleep attacks n (%)**	3 (16.6)	1 (5.3)	0 (0.0)	0 (0.0)	0.242	0.233	NA
**RBDSQ score (n)**	3.1 ± 2.2	3.7 ± 2.6	2.7 ± 2.7	1.1 ± 1.9	0.395	0.648	0.072

Data are presented as mean, standard deviation, number and percentage.

**ESS:** Epworth Sleepiness Scale; **IPD**: Idiopathic Parkinson Disease; **IRLSGRS**: International Restless Legs Syndrome Study Group Scale; **LRRK2-PD**: LRRK2 Parkinson Disease; **NA**: Not applicable; **NMC**: Non-manifesting carriers; **NMNC:** Non-manifesting non-carriers; **PSQI**: Pittsburg Sleep Quality Index; **PD**: Parkinson disease; **PDSS-2**: Parkinson’s Disease Sleep Scale 2; **RBD**: REM sleep behavior disorder; **RBDSQ**: RBD screening questionnaire.

**Table 3:** Polysomnography and Multiple Sleep Latency Test data and comparisons between groups.

	LRRK2-PD(n = 18)	IPD(n = 19)	NMC(n = 17)	NMNC(n = 14)	*p* value(LRRK2-PD vs. IPD)	*p* value(LRRK2-PD vs. NMC)	*p* value(NMC vs. NMNC)
**Age (years)**	61.0 ± 11.2	63.1 ± 11.2	44.4 ± 12.3	50.8 ± 16.0	0.996	<0.001	0.557
**Total sleep time (min)**	362.8 ± 95.8	396.2 ± 81.3	411.4 ± 65.7	409.2 ± 49.5	0.174	0.107	0.471
**Sleep efficiency (%)**	75.0 ± 19.2	82.2 ± 16.0	83.9 ± 13.2	83.5 ± 11.0	0.187	0.177	0.498
**Wake time after sleep onset (min)**	75.6 ± 66.7	70.2 ± 63.5	47.4 ± 37.0	54.1 ± 40.8	0.539	0.154	0.777
**N1 (%)**	15.2 ± 6.9	11.2 ± 6.1	18.1 ± 20.3	12.7 ± 4.0	0.047	0.583	0.896
**N2 (%)**	67.3 ± 9.2	62.2 ± 12.4	52.9 ± 13.4	59.3 ± 8.1	0.136	0.001	0.190
**N3 (%)**	6.9 ± 6.9	10.0 ± 8.3	14.1 ± 7.1	10.8 ± 5.5	0.195	0.006	0.206
**REM sleep (%)**	10.6 ± 7.4	16.5 ± 9.9	18.5 ± 6.1	17.2 ± 6.5	0.059	0.003	0.541
**Arousal Index (n)**	18.5 ± 13.4	19.2 ± 12.5	15.0 ± 11.1	18.1 ± 8.6	0.953	0.428	0.222
**AHI (n)**	9.0 ± 11.6	15.3 ± 15.4	10.0 ± 14.7	14.7 ± 17.8	0.054	0.826	0.485
**AHI ≥15 n (%)**	5 (27.7)	8 (42.1)	4 (23.5)	5 (35.7)	0.428	1.000	0.700
**PLMSI (n)**	7.2 ± 13.3	8.0 ± 13.4	3.3 ± 5.3	4.9 ± 7.6	0.274	0.284	0.835
**PLMSI ≥15 n (%)**	4 (22.2)	4 (21.0)	1 (5.8)	3 (21.4)	1.000	0.346	0.330
**REM sleep behavior disorder (%)**	3 (16.6)	10 (52.6)	0 (0.0)	0 (0.0)	0.037	0.224	1.000
**REM sleep 3- secmini-epochs (n)**	825.5 ± 584.7	909.9 ± 583.2	1264.3 ± 534	1079.4 ± 363	0.663	0.033	0.289
**SINBAR EMG activity (%)**	12.2 ± 14.9	42.2 ± 25.6	8.7 ± 4.8	9.7 ± 7.9	<0.001	0.482	0.757
**Mentalis EMG activity (%)**	10.1 ± 12.1	29.1 ± 24.1	5.5 ± 3.5	5.4 ± 3.8	0.009	0.169	0.958
**Mentalisplus AT EMG activity (%)**	15.4 ± 15.6	41.4 ± 23.7	14.5 ± 8.9	8.5 ± 4.9	0.007	0.352	1.000
**MSLT sleeplatency (min)**	6.5 ± 4.6	8.0 ± 4.9	9.3 ± 5.3	7.2 ± 4.3	0.334	0.119	0.371
**MSLT sleep latency <5min n (%)**	7 (41.2)*	8 (42.1)	4 (23.5)	4 (28.6)	0.955	0.472	0.700
**MSLT sleep latency < 8min n (%)**	12 (70.6)*	8 (42.1)	6 (35.3)	10 (71.4)	0.106	0.153	0.466
**SOREMPs on MSLT n (%)**	1 (5.8)*	1 (5.3)	0 (0.0)	1 (7.1)	1.000	1.000	0.483

Data are presented as mean, standard deviation, number and percentage.

*MSLT was performed in 17 out of 18 LRRK2-PD patients since one refused to undergo this test. Percentages on MSLT measures are therefore based on these 17 subjects.

**AHI**: Apnea-hipopnea index; **LRRK2-PD**: LRRK2 Parkinson disease; **Mentalis**: polysomnographic montage quantifying "any" (phasic and tonic) type of EMG activity in the mentalis muscle during REM sleep, using 3-sec mini-epochs (the cut-off for patients with RBD is 18%)[38]; **Mentalis plus AT**: polysomnographic montage quantifying "any" (phasic and tonic) type of EMG activity in the mentalis muscle plus phasic EMG activity in the right and left anterior tibialis during REM sleep, using 3-sec mini-epochs (the cut-off for patients with RBD is 46.4%) [38]; **MSLT**: Multiple Sleep Latency Test; **NMC**: non-manifesting carriers; **NMNC**: non-manifesting non-carriers; **NA**: Not applicable; **PLMS**: Periodic Leg Movements in Sleep; **PLMSI**: Periodic Leg Movements in Sleep Index; **RBD**: REM Sleep Behavior Disorder; **SINBAR**: polysomnographic montage quantifying "any" (phasic or tonic) type of EMG activity in the mentalis muscle and phasic EMG activity in the right and left flexor digitorumsuperficialis muscles during REM sleep, using 3-sec mini-epochs (the cut-off for patients with RBD is 32%)[38]; **SOREMPs**: Sleep Onset REM Sleep Periods.

### Sleep in LRRK2-PD subjects

There were nine women and nine men with a mean age of 61.0 ± 11.2 years. Fourteen (78%) subjects had PSQI ≥6 and eight (44%) a PDSS-2 ≥15, which are indicative of subjective poor sleep quality. Six (33%) patients complained of sleep onset insomnia, ten (56%) of sleep fragmentation, nine (50%) of nocturnal akinesia, 11 (61%) of nocturia, and seven (39%) of early awakening. Sleep onset insomnia correlated with HADS total score (r = 0.512, p = 0.003), HADS depression score (r = 0.707, p = 0.001), PSQI score (r = 0.740, p<0.001) and PDSS-2 score (r = 0.688, p = 0.002).

Six (33%) patients complained of EDS andnone of them reported that this symptom antedated the onset of motor symptoms. Of these six patients, four had an ESS score ≥11. MSLT was performed in 17 LRRK2-PD subjects and 12(71%)(six women and six men, 11 carrying the G2019S mutation and one the R1441G mutation) showed a mean sleep latency of ≤8 minutes on the MSLT, which is indicative of objective EDS. Only four of these 12 patients with abnormal sleep latency on the MSLT complained of EDS. There was no correlation between the mean sleep latency on the MSLT with any demographic, clinical and V-PSG variable such as age, PD duration and severity, therapy with dopaminergics, antidepressants and benzodiazepines, pain, HADS score, PSQI score, PDSS-2 score, sleep complaints, body mass index, nocturnal total sleep time, sleep efficiency, PLMI and AHI.

Three (17%) patients, two women and one man, reported sleep attacks occurring several years after the onset of parkinsonism. One patient reported a temporal association between the introduction of pramipexol and the onset of sleep attacks. His sleep efficiency on the V-PSG was 93%, no apneas were detected, and the mean sleep latency on the MSLT was 1 min. Another patient with sleep attacks was taking L-Dopa, pramipexol and alprazolam, the sleep efficiency in the V-PSG was 70%, AHI was 19 and sleep latency on the MSLT was 4 min. The remaining patient experiencing sleep attacks was receiving L-Dopa, pramipexol and escitalopram, sleep efficiency in the V-PSG was 91%, AHI was 25, and sleep latency on the MSLT was 5 min.

None of the LRRK2-PD patients had a narcoleptic phenotype. None had two SOREMPs on the MSLT. A single SOREMP was detected in one subject who was sleep and REM sleep deprived (nocturnal PSG showed a sleep efficiency of 30%and REM sleep was not recorded).

Three (17%) unrelatedG2019SLRRK2-PD had RLS (Table 4). RLS severity was mild in one patient, moderate in one and severe in one. One patient reported that RLS symptomatology started before and two after parkinsonism onset.

**Table 4:** Subjects with restless legs syndrome.

Subject	LRRK2-PD-03	LRRK2-PD-09	LRRK2-PD-14	IPD-14	IPD-15	IPD-16	NMC-05	NMC-08	NMNC-04	NMNC-09
**Clinical status**	LRRK2 PD	LRRK2 PD	LRRK2 PD	IPD	IPD	IPD	NMC	NMC	NMNC	NMNC
**LRRK2 mutation**	G2019S	G2019S	G2019S	No	No	No	G2019S	R1441C	R1441G	G2019S
**Age (years)**	66	77	64	73	75	51	47	61	34	58
**Gender**	Male	Female	Female	Female	Male	Male	Male	Female	Male	Female
**Age at parkinsonism onset (years)**	64	65	56	69	69	44	NA	NA	NA	NA
**Antiparkinsonian drugs**	No	L-dopa, pramipexole, rasagiline	L-dopa, pramipexole, rasagiline	L-dopa, pramipexole	L-dopa, entacapone	Ropinirole	No	No	No	No
**LEDD (mg)**	0	1310	805	775	399	140	0	0	0	0
**Age at RLS onset (years)**	25	70	59	69	69	20	20	56	29	56
**RLS Family History**	No	No	No	No	No	Yes	Yes	No	No	No
**IRLSGRS score (n)**	21	8	18	14	12	2	15	11	0	23
**ESS score (n)**	5	7	17	11	8	8	12	4	4	4
**PSQI score (n)**	13	10	15	11	12	6	3	5	2	18
**PDSS-2 score (n)**	33	15	30	13	16	4	22	10	2	21
**Subjective sleep complaints**	Sleep onset insomnia, sleep fragmentation	Sleep fragmentation, early awakening	Sleep onset insomnia, sleep fragmentation	Sleep fragmentation, early awakening	Sleep fragmentation, early awakening	No	Sleep fragmentation	Sleep fragmentation	No	Sleep onset insomnia, sleep fragmentation, early awakening
**PLMSI (n)**	26	43	0	10	15	4	19	10	17	21
**Sleep efficiency (%)**	71	70	91	79	35	92	88	65	91	71
**Mean Sleep Latency onset on MSLT (min)**	14	4	5	12	18	2	5	9	4	13

**ESS**: Epworth Sleepiness Scale; **IRLSGRS**: International Restless-legs syndrome Study Group Scale; **LEDD**: L-Dopa Equivalent daily dose; **LRRK2-PD**: LRRK2 Parkinson disease subjects;**MSLT**: Multiple Sleep Latency Sleep Test; **NA**: Not applicable; **NMC**: Non-manifesting carriers; **NMNC**: Non-manifesting non-carriers; **PSQI**: Pittsburg Sleep Quality Index; **PDSS-2**: Parkinson Disease Sleep Scale 2; **PLMSI**: Periodic Leg Movements in Sleep Index; **RLS:** Restless Legs Syndrome.

In three patients REM sleep was not recorded during V-PSG (none of these three subjects reported a history of dream-enacting behaviors suggestive of RBD). Three (17%) unrelated LRRK2-PD G2019S carriers had RBD (Table 5). All three were men and had thepostural instability and gait difficulty motor subtype. In two, the onset of RBD was reported to occur 10 and 12 years after the onset of parkinsonism, respectively. These two patients were unaware of displaying dream-enacting behaviors that were reported by their wives who did not link the onset of RBD symptomatology with the introduction of any medication. The remaining RBD patient had no bed partner, and he reported no dream recall and lack of abnormal sleep behaviors. However, V-PSG was demonstrative of RBD showing excessive EMG activity in REM sleep linked to prominent body jerking and talking. One additional patient reported talking, laughing and kicking and recalled dreams of being attacked and arguing with a RBDSQ score of 8 points (which is suggestive of RBD). In this patient, however, V-PSG showed normal muscle atonia in REM sleep (SINBAR EMG activity of 10%)and therefore RBD was ruled out[38]. V-PSG showed AHI of 65. Videotape analysis disclosed gesturing and talking that only occurred during arousals at the end of obstructive apneic events during REM sleep and to a lesser extent during no-REM sleep. We concluded that this patient had severe obstructive sleep apnea mimicking RBD symptomatology (“pseudo-RBD”)[40].

**Table 5:** LRRK2-Parkinson Disease patients with associated REM sleep behavior disorder.

Patient	LRRK2-PD-2	LRRK2-PD-6	LRRK2-PD-10
**Age (years), gender**	56, male	75, male	71, male
**LRRK2 mutation**	G2019S	G2019S	G2019S
**Age at RBD onset (years)**	Unknown	74	68
**Age at parkinsonism onset (years)**	40	62	58
**UPDRS-III score (n)**	6	10	18
**HoehnandYahr stage (n)**	2	2.5	3
**Motor subtype**	PIGD	PIGD	PIGD
**Falls / Freezing**	Yes / No	Yes / No	Yes / Yes
**Motor fluctuations**	Yes	Yes	Yes
**LEDD (mg)**	664	1674	1940
**MoCA (n)**	28	25	22
**Hallucinations**	No	No	No
**RBDSQ (n)**	2	8	4
**Unpleasant dream recall**	No	Attacked and chased by someone, attacked by animals	No
**Presence of a bed partner**	No	Yes	Yes
**Patient awareness of dream-enacting behaviours**	No	No	No
**Reported dream-enacting behaviors by the bed partner**	No	Talking, screaming, biting the bedpartner	Punching, kicking, knocking off the nightstand, talking, screaming, moaning
**REM sleep behaviors detected during V-PSG**	Prominent limb jerking, talking	Prominent body jerking, kicking	Prominent limb jerking, screaming and moaning
**SINBAR EMG activity (%)**	39.8	42.1	35.9
**M + AT EMG activity (%)**	39.4	42.8	36.8
**Mentalis EMG activity (%)**	28.3	37.4	32.4

**LEDD**: L-Dopa Equivalent daily dose; **M+AT**: polysomnographic montage quantifying "any" (tonic or phasic) type of EMG activity in the mentalis muscle plus bilateral anterior tibialis phasic EMG activity in REM sleep, using 3-sec mini-epochs (the cut-off for patients with RBD is 46.4%)[38]; **Mentalis**: EMG activity quantification of "any" (tonic and phasic) type of EMG activity in the mentalis muscle during REM sleep, using 3-sec mini-epochs (the cut-off for patients with RBD is 18.2%.)[38]; **MoCA**: Montreal Cognitive Assessment; **PIGD**: postural instability gait difficulty motor subtype; **SINBAR**: polysomnographyc montage quantifying "any" (tonic or phasic) type of EMG activity in the mentalis muscle and phasic EMG activity in the right and left flexor digitorumsuperficialis muscles during REM sleep, using 3-sec mini-epochs (the cut-off for patients with RBD is 32%)[38]; **RBD**: REM sleep behaviour disorder; **RBDSQ**: REM sleep behavior disorder screening questionnaire; **UPDRS-III**: Unified Parkinson Disease Rating Scale motor exam; **V-PSG:** video-polysomnography.

The LRRK2-PD patient carrying theR1441G mutation complained of early awakening but he had no subjective EDS, no sleep attacks, no RLS, no RBD, and V-PSG showed sleep efficiency of 95%, absence of apneas and PLMS, and sleep onset latency on the MSLT of 4 min without SOREMPs.

### Sleep in LRRK2-PD vs. IPD subjects

Compared to IPD, LRRK2-PD patients reported more frequently sleep onset insomnia (p = 0.005), had greater (worse) PSQI score (p = 0.033) and PSQI ≥6 (p = 0.025), more benzodiazepines usage (p = 0.003) and lower UPDRS-III score (p = 0.022). In a secondary analysis controlled for benzodiazepine and dopamine agonist intake, UPDRS-III score (p = 0.012), PSQI ≥6 (p = 0.012), and PSQI and score (p = 0.056) were still significant but not sleep onset insomnia (p = 0.998).

There were no differences between LRRK2-PD and IPD groups in percentage of patients complaining of EDS, ESS score, mean sleep latency on the MSLT, and percentage of patients with mean sleep latencies shorter than five and eight min on the MSLT.

**Fig 1:**
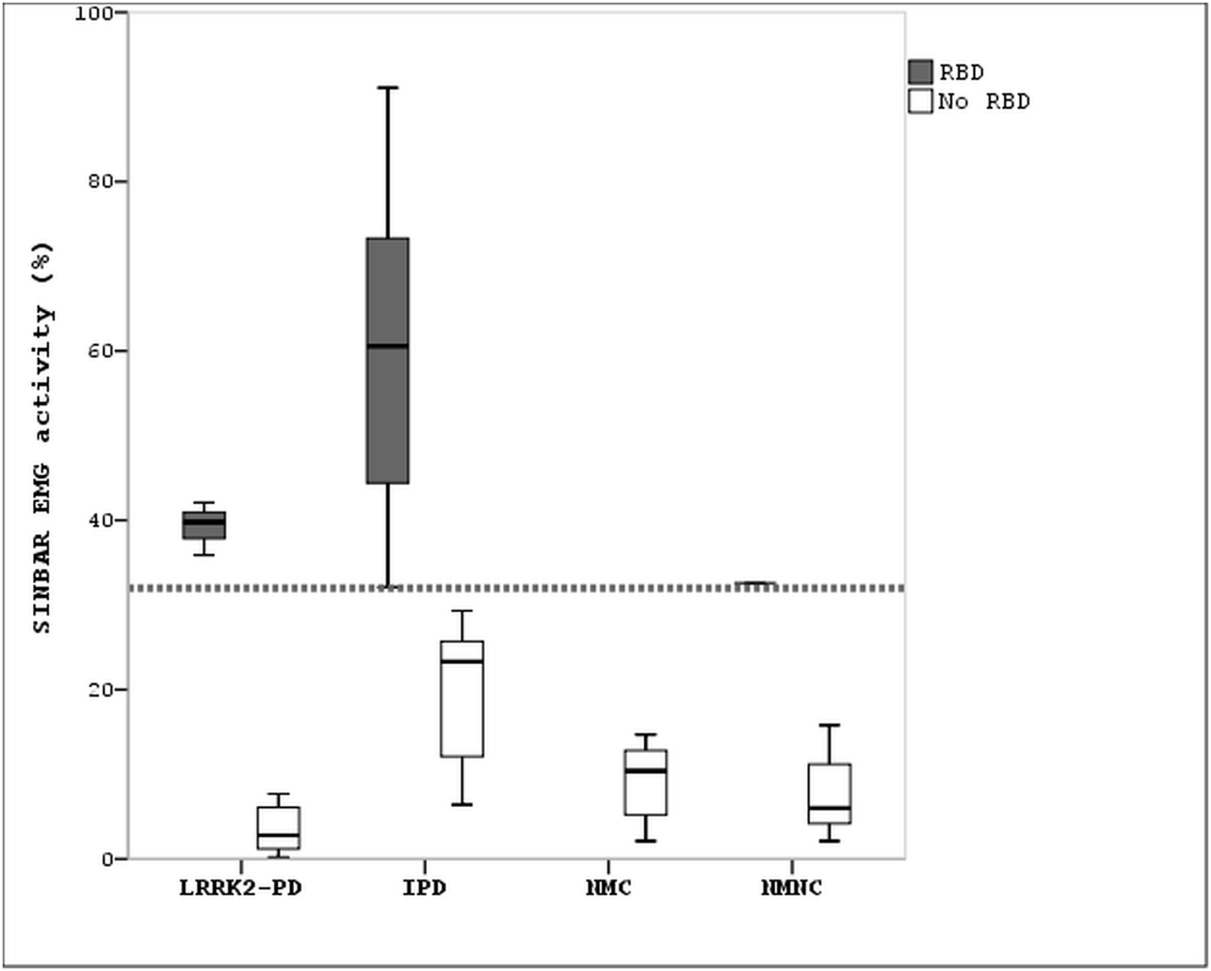
Electromyographic activity in REM sleep. Bars represent the percentage of electromyographic activity found during REM sleep in the different groups included in the study. Grey bars represent the percentage of electromyographic activity found in individuals who were diagnosed with RBD. White bars represent the percentage of electromyographic activity found in individuals who had no RBD. The dotted transversal line indicates that 32% of electromyographic activity is the cut-off value for the diagnosis of RBD according to the SINBAR method. Electromyographic activity ≥32% during REM sleep is indicative of RBD[38]. IPD: Idiopathic Parkinson disease; LRRK2-PD: LRRK2 Parkinson disease; NMC: non-manifesting carriers; NMNC: non-manifesting non-carriers; RBD: REM sleep behavior disorder; SINBAR: polysomnographic montage quantifying "any" (phasic or tonic) type of EMG activity in the mentalis muscle and phasic EMG activity in the right and left flexor digitorum superficialis muscles during REM sleep using 3-sec mini-epochs.

Overall, the LRRK2-PD group had less EMG activity in REM sleep than the IPD group (Fig 1). RBD was diagnosed in three (17%) patients with LRRK2-PD and in ten (53%) with IPD (p = 0.034) (Tables 5 and 6). Among the patients with RBD, those with LRKK2-PD had less EMG activity in REM sleep than those with IPD (Fig 1). Seven IPD patients with true RBD had a RBDSQ score less than seven (which is suggestive of not having RBD according to this instrument). One IPD patient reporting dream-enacting behaviours and nightmares had a RBDSQ score of eight points (suggestive of RBD), but V-PSG demonstrated that he had not RBD but severe obstructive sleep apnea (AHI = 69)mimicking this parasomnia (“pseudo RBD”)[40].

**Table 6:** Patients with idiopathic Parkinson Disease associated with REM sleep behavior disorder.

Patient	IPD-01	IPD-02	IPD-04	IPD-06	IPD-08	IPD-010	IPD-14	IPD-18	IPD-19	IPD-20
**Age (years), gender**	57, female	69, male	75, male	60, male	62, male	63, female	73, female	72, male	78, female	56, female
**Age at RBD onset (years)**	32	59	73	54	48	56	65	Unknown	75	56
**Age at parkinsonism onset (years)**	42	64	74	49	56	56	68	62	65	52
**UPDRS-III (n)**	20	28	23	9	24	30	26	44	16	24
**Hoehn and Yahr stage (n)**	2	2	1	2	2.5	2	3	2.5	2	1.5
**Motor subtype**	Indeterminate	TD	TD	PIGD	PIGD	PIGD	PIGD	PIGD	PIGD	TD
**Falls / Freezing**	No / No	No / No	No / No	Yes / No	No / No	No / No	No / Yes	No / Yes	Yes / No	No / No
**Motor fluctuations**	Yes	No	No	Yes	Yes	Yes	No	No	Yes	No
**LEDD (mg)**	410	120	100	773	900	700	775	960	1597	0
**MoCA (n)**	ND	ND	22	26	24	25	ND	ND	ND	ND
**Hallucinations**	No	No	No	Yes	No	No	Yes	No	Yes	No
**RBDSQ (n)**	3	2	5	4	7	3	9	2	8	2
**Unpleasant dream recall**	Falling from a cliff	Arguing with someone, attacked by someone, falling from a cliff	No	No	Attacked by someone, falling from a cliff	Falling from a cliff, being robbed	No	No	Arguing with someone, chased by someone, children being kidnapped	No
**Presence of a bedpartner**	Yes	Yes	Yes	Yes	Yes	Yes	Yes	Yes	Yes	Yes
**Patient awareness of dream-enacting behaviours**	No	Yes	No	No	No	No	No	No	Yes	No
**Reported dream-enacting behaviorsby the bed partner**	Gesturing, screaming, talking, laughing	Gesturing, punching, knocking off the nightstand, screaming	Gesturing, punching, talking	Gesturing, punching kicking, screaming, talking, laughing	Gesturing, screaming, talking, laughing	Talking, kicking	Gesturing,punching, kicking, knocking off the nightstand, talking, screaming, laughing, crying	No	Gesturing, punching, kicking, knocking off the nightstand, creaming, talking, crying, laughing, falling out of bed causing lacerations	Screaming
**REM sleep behaviors detected during V-PSG**	Prominent limb and body jerking, moaning	Prominent limb and body jerking, gesturing, punching	Limb and body jerking, talking moaning	Prominent body jerking, gesturing	Prominent limb jerking	Prominent body jerking, gesturing	Talking, moaning, swearing, prominent, body jerking, punching	Prominent limb and body jerking	Prominent limb jerking, murmuring	Prominent limb jerking, kicking and punching
**SINBAR EMG activity (%)**	41.7	91.1	58.6	63.8	32.1	44.4	85.3	62.5	73.3	49.6
**M + AT EMG activity (%)**	38.9	84.4	53.8	66.3	33.1	42.3	76.8	63.3	68.1	54.0
**Mentalis EMG activity (%)**	21.7	83.4	37.7	43.5	6.9	38.3	72.0	35.4	58.7	43.7

**LEDD:** L-Dopa Equivalent daily dose; **M+AT**: polysomnographic montage quantifying "any" (tonic or phasic) type of EMG activity in the mentalis muscle plus bilateral anterior tibialis phasic EMG activity in REM sleep, using 3-sec mini-epochs (the cut-off for patients with RBD is 46.4%)[38]; **Mentalis**: EMG activity quantification of "any" (tonic and phasic) type of EMG activity in the mentalis muscle during REM sleep, using 3-sec mini-epochs (the cut-off for patients with RBD is 18.2%[38]; **MoCA**: Montreal Cognitive Assessment; **ND**: not done; **PIGD:** postural instability gait difficulty motor subtype; **SINBAR**: polysomnographyc montage quantifying "any" (tonic or phasic) type of EMG activity in the mentalis muscle and phasic EMG activity in the right and left flexor digitorumsuperficialis muscles during REM sleep, using 3-sec mini-epochs (the cut-off for patients with RBD is 32%[38]; **RBD**: REM sleep behaviour disorder; **RBDSQ:** REM sleep behavior disorder screening questionnaire; **TD**: tremor dominant motor subtype; **UPDRS-III**: Unified Parkinson Disease Rating Scale motor exam; **V-PSG**: video-polysomnography.

### Sleep in NMC of *LRRK2* mutations

Subjects were nine women and eight men with a mean age of 44.4 ± 12.3 years. Sleep complaints in this group were not common. Only one subject had a PSQI ≥6 and two a PDSS-2 ≥15. Two complained of sleep onset insomnia, four of sleep fragmentation, one of early awakening, two had RLS, and only one used benzodiazepines.

Four (23%) subjects complained of EDS and all four had an ESS score ≥11. Sleep attacks were not reported by any individual. The mean sleep latency on the MSLT was in the normal range (9.3 ± 5.3 min) and SOREMPs did not occur. The mean sleep latency on the MSLT was not correlated with any demographic, clinical, and PSG variable.

None of the NMC had RBD. One NMC with RLS reported dream-enacting behaviors (e.g., kicking, gesturing, talking) and nightmares (e.g., being attacked by animals, arguing with people), and had a RBSQ score of 9points (suggestive of RBD). However, V-PSG detected normal muscle atonia in REM sleep (SINBAR EMG activity of 10.7%) excluding RBD[38]. V-PSG showed a PLMSI of 29 where PLMS were associated with arousals and videotape analysis showed that the leg movements also involved the trunk and upper limbs resulting in prominent body jerking associated with arousals. We concluded that in this subject the RBD-like symptomatology was likely to be secondary to PLMS (“pseudo-RBD”)[41].

### Sleep in LRRK2-PD vs. NMC of *LRRK2* mutations

LRRK2-PD patients were older and had higher UPDRS-I-IV scores, HADS depression subscore, early awakening, PSQI score, PSQI ≥6, PDSS-2 score and more benzodiazepine intake. In PSG, LRRK2-PD subjects had higher percentage of N2, and lower percentages of N3 and REM sleep than NMC.

Six LRRK2-PD patients and three NMC complained of EDS, and the mean ESS score was similar between both groups. The mean sleep latency was shorter in the LRRK2-PD than in NMC group but difference was not statistically significant (6.5 ± 4.6 vs. 9.3 ± 5.3 min; p = 0.119). RBD was diagnosed in three LRRK2-PD patients and in none NMC.

### Sleep in NMC vs. NMNC of *LRRK2* mutations

Demographic, clinical, PSG and MSLT variables were similar between these two groups.

## Discussion

To the best of our knowledge, this is the first systematic study evaluating sleep disorders with subjective measures (comprehensive sleep interview, validated sleep scales and questionnaires) and objective tools (nocturnal V-PSG followed by MSLT) in *LRRK2* mutation carriers. The study evaluated individuals who had developed motor symptoms(LRRK2-PD and IPD) and NMC who are at risk to manifest PD. Despite the limited number of subjects included, we believe that our findings are relevant considering the prevalence of LRRK2 mutation in the general population[9, 11]. Subjects were not selected on the basis of any specific sleep complaint or previous diagnosis of a sleep disorder. We found that sleep complaints were frequent in LRRK2-PD and included sleep onset insomnia, sleep fragmentation and early awakening. Short sleep onset latencies on the MSLT, indicative of objective EDS, were frequent. Sleep attacks occurred in three LRRK2-PD patients and a narcoleptic phenotype was not observed on the MSLT. RBD occurred in three LRRK2-PD patients. EDS and RBD were reported to start after onset of parkinsonism. RLS, PLMS and obstructive sleep apnea were not prominent features in LRRK2-PD. In NMC, EDS was rarely reported and RBD was absent, suggesting that these sleep disorders are not premotor markers of LRRK2-PD.

Mutations in the *LRRK2* gene are the most common cause of monogenic PD[11]. In Spain, the most frequent *LRRK2* mutations are G2019S and R1441G mutations, the latter particularly in the Basque Country (Northern Spain)[9, 13–14]. The motor phenotype of LRRK2-PD resembles IPD and is characterized by parkinsonism of late onset that responds to L-Dopa therapy and motor complications of dopaminergic therapy[11]. Nonmotor symptoms are also frequent in LRRK2-PD although they may differ when compared to IPD. Olfactory dysfunction, constipation, cardiac sympathetic denervation and neuropsychological impairment are less prevalent and less severe in LRRK2-PD than in IPD [13, 15, 42]. In contrast, depression and worse color discrimination have been reported to occur more frequently in LRRK2-PD[12, 43].

Previous studies reported that LRRK2-PD patients may complaint of sleep onset insomnia, repeated awakenings and features suggestive of RBD[11, 13, 15, 43–45]. However, these studies did not employ scales or questionnaires specifically designed to examine sleep, and V-PSG and MSLT were not used to identify objectively sleep disorders like EDS and RBD. Overall, we have found the same sleep disorders in LRRK2-PD than those previously described in IPD (sleep onset insomnia, fragmented sleep, early awakenings, EDS, sleep attacks and RBD) but with a distinct pattern; sleep onset insomnia was more frequent, EDS was similar and RBDwas less frequent and less severe in LRRK2-PD than in IPD.

Compared to IPD, sleep onset insomnia was more frequent in LRRK2-PD and it was associated with depressive symptoms and poor sleep quality. Of note, sleep onset insomnia in LRRK2-PD was not linked to features such as age, gender,PD severity and duration, anxiety, and cognitive status. These findings may suggest that difficulty falling asleep in LRRK2-PD may be explained in part by depression. Our findings are consistent with other studies in LRRK2-PD where sleep disorders, mainly sleep onset insomnia and repeated awakenings, occurred in 65% of the cases[43]and they were related to depression[12, 43, 45].

EDS was investigated through subjective (sleep interview and ESS) and objective (MSLT) measures[46–47]. All LRRK2-PD patients who complained of experiencing EDS reported that parkinsonism preceded the onset of this symptom. MSLT in LRRK2-PD showed that an increased level of daytime sleepiness is a common feature that is frequently misperceived by patients themselves. The finding that subjects were unaware of being abnormally asleep has also been reported in 13–47% IPD patients without complaints of EDS[48–50].

In LRRK2-PD, demographic, clinical and sleep variables did not distinguish between subjects with abnormal and normal sleep latencies on the MSLT. Potential causes of EDS such as PD duration and severity, pain, depressive symptoms, the use of benzodiazepines, antidepressants or dopaminergic agents, and obstructive sleep apnea failed to reveal a relationship with reduced sleep latency on the MSLT. This suggests that the origin of EDS in LRRK2-2 PD is an intrinsic feature of the disease itself related to an abnormal sleep-wake control, and it is not the result of nocturnal sleep abnormalities, parkinsonism, or any drug.

Sleep attacks were noticed by three subjects with LRRK2-PD. As previously described in IPD, sleep attacks in our LRRK2-PD patients were linked to the introduction of a dopamine agonist, obstructive sleep apnea or an increasedlevel of daytime sleepiness on the MSLT[3, 51]. In contrast to what has been reported in IPD[48, 51], SOREMPs were not common in *LRRK2* mutation carriers. We did not identify a narcoleptic phenotype in any patient carrying *LRRK2* mutations.

Three LRRK2-PD patients had RBD confirmed by V-PSG. The pathophysiology of RBD is related to dysfunction of the brainstem nuclei that regulate REM sleep atonia, namelythe locus subcoeruleus and the magnocellularis nucleus[52]. Postmortem studies have shown that these nuclei are severely damaged in IPD associated with RBD[53]. To the best of our knowledge the state of these nuclei has not been reported in LRRK2-PD postmortem reports[54]. Our study showed that compared to RBD occurring in IPD, RBD in LRRK2-PD was less frequent and had less EMG activity during REM sleep. Thus, it can be speculated that damage of the brainstem structures that regulate REM sleep atonia is less marked in LRRK2-PD than in IPD. This might be attributed to the heterogeneous neuropathological substrate found in LRRK2-PD[54].

All three LRRK2-PD patients with RBD were men, carried the G2019S mutation, had the postural instability and gait difficulty motor subtype, no hallucinations, no cognitive impairment and lack of a previous history of impulse control disorders. RBD symptomatology developed several years after parkinsonism onset. All were unaware of displaying dream-enacting behaviors that were described by their bed partners, a finding which is also common in idiopathic RBD and RBD secondary to IPD and multiple system atrophy[53].

The diagnosis of RBD was based on clinical and V-PSG criteria showing excessive EMG activity in REM sleep associated with vigorous behaviors. Only V-PSG was able to identify true RBD patients that were unaware of displaying dream-enacting behaviors (false negatives) and to exclude RBD in subjects with other sleep disorders (severe obstructive sleep apnea and prominent PLMS involving the trunk and upper limbs) who presented with dream-enacting behaviors and nightmares (false positives, RBD mimics). This reflects that V-PSG is necessary to diagnose RBD and that questionnaires, particularly when self-administrated, may be not adequate.

The penetrance of LRRK2 mutations is incomplete and increases in an age dependent manner[11]. In *LRRK2* mutation carriers mean age of PD diagnosis is 58 years, and the estimated risk to develop PD ranges from 12% to 36% at 59 years,26% to 59% at 69 years, and 47% to 80% at 79 years[11, 55]. This indicates that asubstantial proportion of NMC with increased age could bein the prodromal stage of LRRK2-PD. The study of NMC provides a unique opportunity to characterize the premotor stage of LRRK2-PD and the search for markers of conversion. Conversion markers in prodromal LRRK2-PD may prove useful when neuroprotective strategies will become available. Available data suggest thathyposmia, depression and constipation can be present in the premotor stage of LRRK2-PD and IPD[1, 7, 10, 12]. RBD is also a marker of premotor IPD[52]. It is uncertain if EDS predicts IPD[4]. Two population-based studies in the elderly showed that subjects who reported EDS had increased risk of developing IPD[56–57]. However, most studies have demonstrated that EDS is uncommon in untreated de novo IPD[58–60]. Our study in *LRRK2* mutation carriers did not show that RBD and EDS may be features of prodromal LRRK2-PD. RBD was not detected in NMC and when present in LRRK2-PD patients the onset of parkinsonism preceded the onset of RBD for several years. The study of sleep disorders therefore does not seem to be useful to distinguishNMC who are in the premotor stage of LRRK2-PD and are likely to develop PD during lifetime. However this assumption should be taken with caution since in our study 1) the number of NMC included in our study was relatively small, 2) the mean age of NMC was only of 44 years and these subjects may still be somewhat far in time from conversion, and 3) clinical follow-up of our cohort is needed. Further studies of a larger NMC cohort with longitudinal follow-up should permit to assess if sleep disorders are markers for phenoconversion to PD.

In summary, our findings show that sleep disorders in LRRK2-PD are common. Sleep onset insomnia was more frequent, EDS was similar and RBD was less frequent and severe in LRRK2-PD than in IPD. RBD and EDS seem not to occur in the premotor stage of LRRK2-PD.
